# Gill monogeneans of Nile tilapia (*Oreochromis niloticus*) and red hybrid tilapia (*Oreochromis* spp.) from the wild and fish farms in Perak, Malaysia: infection dynamics and spatial distribution

**DOI:** 10.1186/s40064-016-3266-2

**Published:** 2016-09-20

**Authors:** Shen-Yin Lim, Ai-Lin Ooi, Wey-Lim Wong

**Affiliations:** 1 Department of Biological Science, Faculty of Science, Universiti Tunku Abdul Rahman, 31900 Kampar, Perak Malaysia; 2 Department of Agricultural and Food Science, Faculty of Science, Universiti Tunku Abdul Rahman, 31900 Kampar, Perak Malaysia

**Keywords:** *Cichlidogyrus*, *Scutogyrus*, *Oreochromis* spp., Infection dynamics, Spatial distribution

## Abstract

Tilapia is one of the commercially important fish in Malaysia as well as in other parts of the world. An understanding of monogenean infection dynamics in tilapia fish may assist us in searching for some intervention measures in reducing the loss of fish caused by parasitic diseases. The present study aimed (1) to compare infection level of monogeneans between the wild and cultured *Oreochromis niloticus*, and between the cultured *O. niloticus* and cultured red hybrid tilapia, and (2) to examine the spatial distribution of monogenean species over the gills of the different host species. From a total of 75 fish specimens, six species of monogeneans from two genera: *Cichlidogyrus* (*C. halli*, *C. mbirizei*, *C. sclerosus*, *C. thurstonae*, *C. tilapiae*) and *Scutogyrus* (*S. longicornis*) were identified. Data showed that the infection level of cultured *O. niloticus* was higher than that of the wild *O. niloticus*, however, the former was lower than that of the cultured red hybrid tilapia. Higher species richness of monogeneans was observed in the cultured red hybrid tilapia as compared to the others. Results for spatial distribution showed that the monogeneans have no preference on the left or right sides of the gills. However, *C. halli*, *C. mbirizei*, and *C. tilapiae* showed preferences on specific gill arches in the cultured *O. niloticus* and red hybrid tilapia. In general, the gill arch IV harboured the least number of monogeneans. The susceptibility of monogenean infection between the different types of tilapia is discussed.

## Background

The growing demand for food sources, particularly protein, has made aquaculture to be one of the fastest growing food sectors in the world. A variety of freshwater fish, such as carp, tilapia and catfish has been cultured in many parts of the world (FAO 2012, 2014) to meet the demands and preferences of consumers. However, the introduction of these fish beyond their native range have caused the co-introduction of parasites along with their hosts to new localities and transmitted to native hosts, causing emergence of new diseases in the native fish (Lymbery et al. 2014).

Tilapia, which is originated from Africa, has become one of the major cultured fish in the world after carp fish (El-Sayed 2006; Wang and Lu 2015). This is because tilapia has the ability to tolerate a wide range of environment conditions, allowing them to be introduced and distributed to many countries outside Africa such as Asia, Southeast Asia, USA and Europe (El-Sayed 2006; Philippart and Ruwet 1982). The tilapia, *Oreochromis mossambicus* Peters, 1852, was first introduced to Malaysia from Indonesia in 1943. Later, *Oreochromis niloticus* Linnaeus, 1785 was introduced in 1979 because of its fast growing features that are suitable for aquaculture (FAO 2004). However, the red hybrid tilapia (*Oreochromis* spp.) have become popular due to customer preference and become the dominant species (>90 %) cultured in Malaysia (Department of fisheries 2013).

Several parasites including the ciliates, *Trichodina* spp., *Ichthyophthirius multifiliis* Fouquet, 1876, and the monogeneans are the most common parasites infecting the tilapia fish (Braccini et al. 2008; Maneepitaksanti and Nagasawa 2012; Paredes-Trujillo et al. 2016; Zago et al. 2014; etc.). Intensive culture of tilapia such as *O. niloticus* facilitates the transmission of these parasites, especially monogeneans that provoke severe epizootic, causing high mortalities of tilapia and economic loss in aquaculture (Akoll et al. 2011).

The taxonomy and biology of monogeneans found in tilapia from Africa were well documented (Muterezi Bukinga et al. 2012; Paperna 1960; Paperna and Thurston 1969; Pariselle and Euzet 2009; Vanhove et al. 2011; etc.). Several studies showed that *Cichlidogyrus* and *Scutogyrus* are the common gill monogeneans found on *O. niloticus* and red hybrid tilapia (Agos 2013; Aguirre-Fey et al. 2015; Akoll et al. 2011; Maneepitaksanti et al. 2013; Tombi et al. 2014). Aguirre-Fey et al. (2015) reported that the infection level of *Cichlidogyrus dossoui* Paperna, 1960, *C. sclerosus* Paperna & Thurston, 1969 and *Scutogyrus* sp. were higher in the red hybrid tilapia (red *O. niloticus* and Pargo—UNAM) compare to their parental species (*O. niloticus* and *O. mossambicus*). However, thus far, the species richness and infection dynamics of other *Cichlidogyrus* species between different fish hosts remain unclear. Tombi et al. (2014) showed that monogeneans colonise four pairs of gills arches of *O. niloticus* in an anterio-posterior direction whilst Agos (2013) reported that monogeneans preferred the median arches of red tilapia. Several studies had also been carried out to examine the relationship between the infection dynamics of monogeneans on *O. niloticus* and red hybrid tilapia with some abiotic factors (Agos 2013; Akoll et al. 2011; Suliman and Al-Harbi 2015; Tombi et al. 2014). The questions arising are whether the same monogenean species will likely to distribute differently over the gills of different species of hosts (*O. niloticus vs* red hybrid tilapia) or over the gills of the same host lived in different conditions. Information on the occurrence, infection dynamics and spatial distribution of gill monogeneans from different fish hosts in different conditions are essential to furnish the information of the interactions of these parasites with their hosts. Therefore, the aims of the present study are (1) to compare the occurrence and infection level of gill monogeneans between the wild and cultured *O. niloticus*, and also between *O. niloticus* and the red hybrid tilapia, and (2) to investigate the spatial distribution of monogeneans over the gill arches for all types of fish hosts.

## Methods

### Collection of fish specimens

A total of 46 Nile tilapia (*O. niloticus*) and 29 red hybrid tilapia (*Oreochromis* spp.) were collected for this study. Out of the 46 specimens of *O. niloticus*, 25 specimens were collected from natural waters in Mambang Diawan, Perak (4.2667° N, 101.1500° E) and the other 21 specimens of *O. niloticus* were collected from a fish farm at Temoh, Perak (4.3500° N, 101.16200° E). Whilst 29 specimens of red hybrid tilapia were obtained from three fish farms: Lawan Kuda (4.4500° N, 101.1667°), Simpang Pulai (4.4667° N, 101.1667° E) and Temoh (4.3500° N, 101.16200° E), Perak. Both cultured *O. niloticus* and red hybrid tilapia were reared separately in different ponds at Temoh fish farm. The water quality of the fish ponds was also determined.

### Examination of fish

Collected fish were euthanised by severing their spinal cord immediately prior to examination (AVMA 2000). The total length and standard length of the fish were measured and recorded. The species of fish host were identified according to Froese and Pauly (2009) and Page and Burr (1991).

### Collection and identification of monogeneans

Individual gill arches were detached, and placed in individual labeled petri dishes filled with distilled water. From the anterior portion of the gill arch below the operculum to the posterior, the four left gill arches were numbered as L I–L IV and the four right gill arches were numbered as R I–R IV. Monogeneans were removed carefully with a fine needle. They were mounted on a microscopic slide under a cover-slip directly in drops of ammonium picrate glycine (Malmberg 1957). The numbers of monogenean in each gill arch were counted under a stereomicroscope (Leica Zoom 2000, Germany). Monogenean species were identified according to the shape and/or size of the sclerotised parts of their haptoral and copulatory organs (Ergens 1981; Muterezi Bukinga et al. 2012; Pariselle et al. 2003) using a compound microscope (Leica CME model, Germany).

### Data analyses

Prevalence, mean intensity as described by Margolis et al. (1982), and index of dispersion, *I* (Variance to mean ratio, where *I* > 1 indicated aggregated data) as described by Poulin (1993) were calculated. Chi square test and Fisher’s exact tests were used to compare the prevalence of monogeneans between host populations. The data obtained in the present study did not fall into a normal distribution after log transformations was performed. Therefore, distribution-free 2-sample bootstrap t tests were used to compare the mean intensity of monogeneans in different host groups (wild *O. niloticus vs* cultured *O. niloticus*; and cultured *O. niloticus vs* red hybrid tilapia) (Rózsa et al. 2000). Non-parametric Mann–Whitney U test was used to compare the distribution of monogeneans between left and right sides of gills, where Kruskal–Wallis analysis of variance (ANOVA) and multiple comparison was used to determine the significant difference between four pairs of gill arches (gill arches numbered I–IV). Data analyses for prevalence, mean intensity, Chi square test, Fisher’s exact test and bootstrap 2-sample *t* test (each with 2,000 replicates) were calculated and performed using the program Quantitative parasitology 3.0 (Rózsa et al. 2000). Since the standard deviations are uninformative in aggregated data (Rózsa et al. 2000), confidential interval (Wald method), and bootstrap bias-corrected and accelerated (BCa) confidential limit (cl) was reported for prevalence and mean intensities, respectively. Non-parametric test were performed using the software package SPSS 20.0. The level of significance is tested at the 5 % level.

## Results

**Fig. 1:**
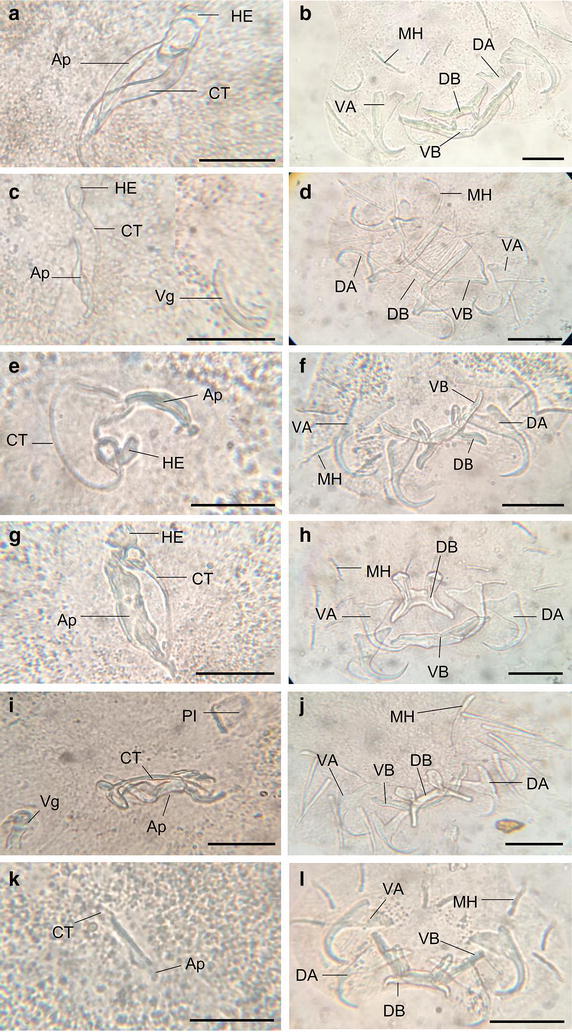
Photomicrographs of the copulatory organs and haptors of *Cichlidogyrus halli* (**a**, **b**), *Scutogyrus longicornis* (**c**, **d**), *C. mbirizei* (**e**, **f**), *C. sclerosus* (**g**, **h**), *C. thurstonae* (**i**, **j**), *C. tilapiae* (**k**, **l**). *Ap* accessory pieces, *CT* copulatory tube, *DA* dorsal anchor, *HE* heel, *MH* marginal hook, *VA* ventral anchor, *DB* dorsal bar, *VB* ventral bar; and *Vg* vagina (*scale bars* 30 μm)

A total of six species of monogeneans were recovered from the gills of studied host specimens (Fig. 1; Table 1). Five species of monogenean belonging to *Cichlidogyrus* (*C*. *halli* Price & Kirk, 1967, *C. mbirizei* Muterezi Bukinga et al., 2012, *C. sclerosus*, *C. thurstonae* Ergens, 1981 and *C. tilapiae* Paperna, 1960) and one to *Scutogyrus* (*Scutogyrus longicornis* Paperna & Thurston, 1969). The monogenean community observed on the gills of the wild and cultured *O. niloticus*, and cultured red hybrid tilapia are different (Table 1). The red hybrid tilapia harbours all the six monogenean species while the wild *O. niloticus* hosts only three monogenean species. The water quality of the natural water was better than that of the fish farms (Table 2).

**Table 1:** Monogenean community on the gills of the wild and cultured *Oreochromis niloticus*, and cultured red hybrid tilapia in the present study

Monogeneans		Fish species		
Family	Species	*Oreochromis niloticus*		Red hybrid tilapia
		Wild	Cultured	Cultured
Dactylogyridae	*Cichlidogyrus halli*		✓	✓
	*C. mbirizei*		✓	✓
	*C. sclerosus*	✓		✓
	*C. thurstonae*		✓	✓
	*C. tilapiae*	✓	✓	✓
	*Scutogyrus longicornis*	✓	✓	✓

**Table 2:** Water quality parameters of the natural water (Mambang Diawan) and three fish farms (Lawan Kuda, Simpang Pulai and Temoh) in Perak

Water parameters	Locations of sampling			
	Mambang Diawan (Mean ± SD)	Lawan Kuda (Mean ± SD)	Simpang Pulai (Mean ± SD)	Temoh (Mean ± SD)
Dissolved oxygen (mg/L)	7.76 ± 2.48	6.80 ± 0.69	7.55 ± 1.02	8.72 ± 0.64
pH	6.90 ± 0.51	7.65 ± 0.22	7.12 ± 0.38	6.53 ± 0.25
Temperature (°C)	25.90 ± 0.14	26.80 ± 0.88	26.30 ± 1.04	26.73 ± 0.25
Ammonia, NH_3_-N (mg/L)	0.15 ± 0.06	0.60 ± 0.00	0.35 ± 0.18	0.42 ± 0.18
Nitrite, NO_2_^—^N (mg/L)	0.01 ± 0.00	0.02 ± 0.01	0.02 ± 0.01	0.01 ± 0.00
Nitrate, NO_3_^−^-N (mg/L)	1.30 ± 0.00	0.90 ± 0.34	1.29 ± 0.34	1.50 ± 0.44

*SD* standard deviation

### Infection level relative to wild and cultured fish

**Fig. 2:**
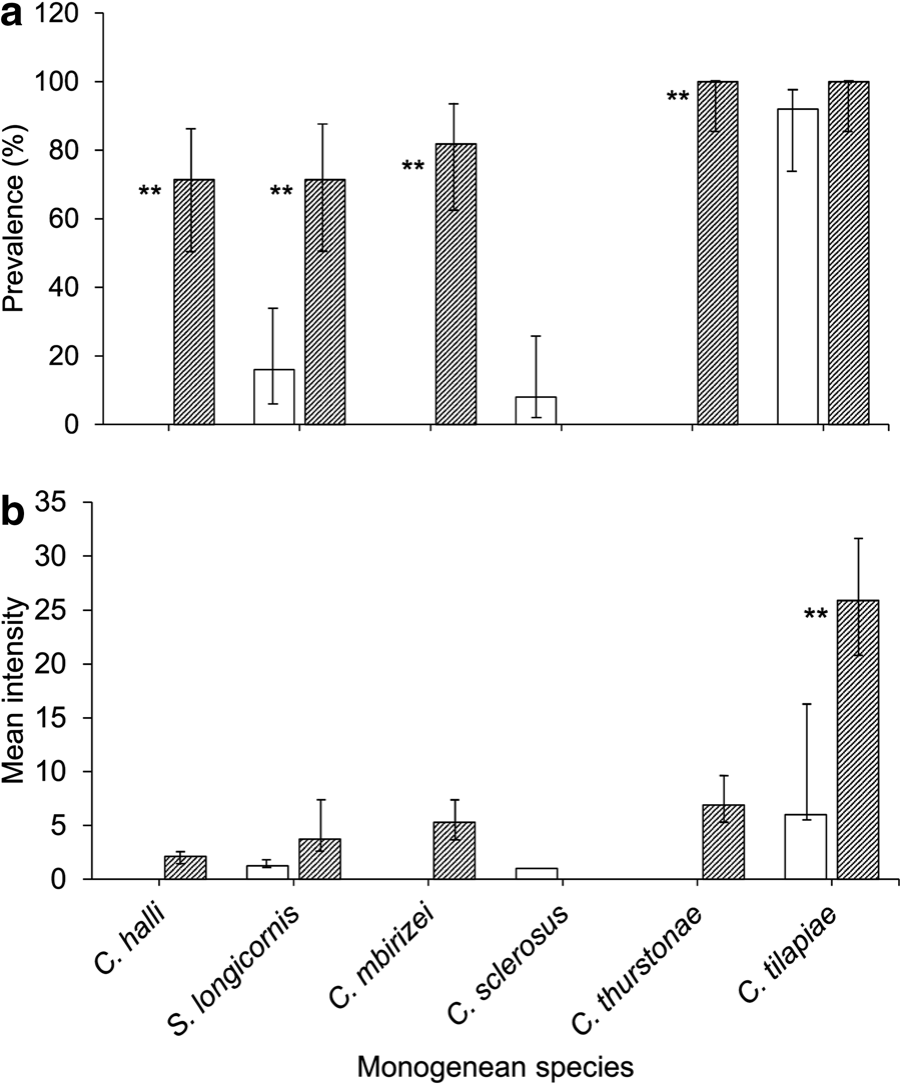
Prevalence (**a**) and mean intensity (**b**) of monogeneans on the gills of wild (n = 25) (*open bar*) and cultured *Oreochromis niloticus* (n = 21) (*dark bar*) (Chi square test; bootstrap 2 sample t test, **p < 0.005)

**Fig. 3:**
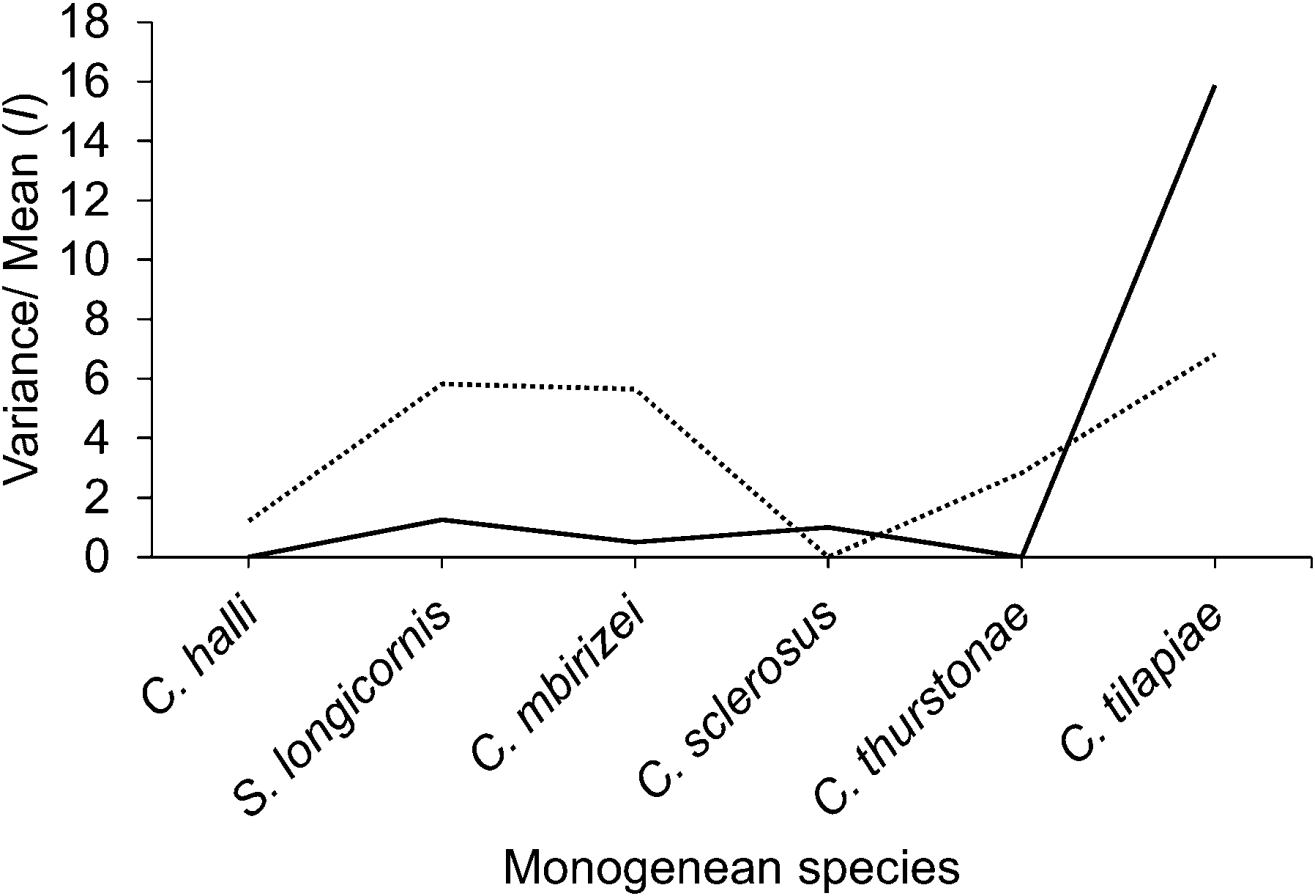
Index of dispersion, *I* (Variance/mean) of the six species of monogeneans on the gills of the wild (*solid line*) and cultured *Oreochromis niloticus* (*dashes line*)

The prevalence and the mean intensity of the monogenean species infecting the gills of the wild (n = 25) and cultured (n = 21) *O. niloticus* were shown in Fig. 2. *Cichlidogyrus tilapiae* was the most frequently observed monogenean on the gills of both the wild and cultured *O. niloticus*, with prevalence of 92 and 100 %, respectively (Fig. 2a). However, the mean intensity of *C. tilapiae* was significantly higher in the cultured *O. niloticus* than in wild *O. niloticus* (t = 6.705, p < 0.001) (Fig. 2b). The prevalence of other monogeneans species in the cultured fish were significantly higher than that of the wild *O. niloticus* except for *C. sclerosus*, which was only infecting the wild tilapia (Fig. 2a). Statistical analysis for the mean intensity of *C. halli*, *C. mbirizei*, *C. thurstonae* and *C. sclerosus* between wild and cultured fish was not performed in the present study because one of the host groups (wild or cultured *O. niloticus*) was not infected by these monogeneans (Fig. 2b). All the six species of monogeneans showed aggregated distribution (*I* > 1) (Fig. 3). However, higher aggregation of monogenean species were found in the cultured fish as compared to the wild fish except for *C. sclerosus* that is only present in the wild fish and for *C. tilapiae*, which aggregated more in the wild fish (Fig. 3).

### Infection level relative to cultured host species

**Fig. 4:**
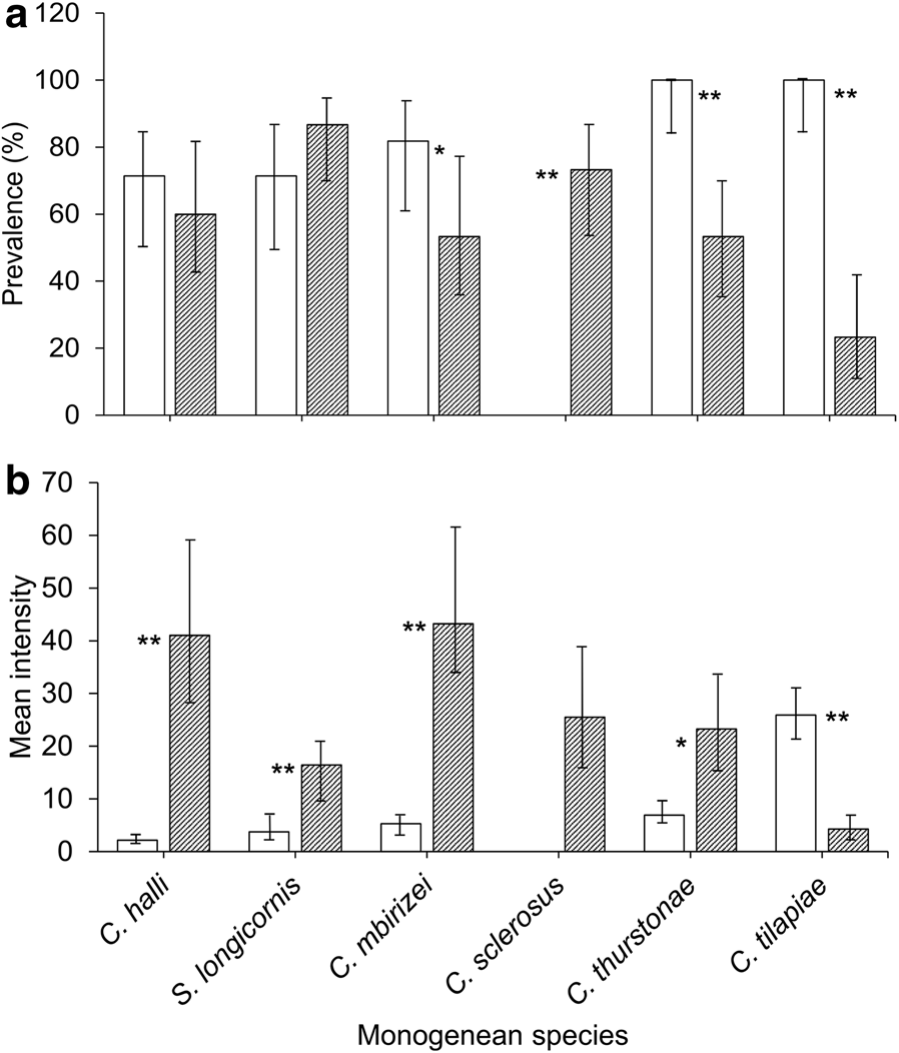
Prevalence (**a**) and mean intensity (**b**) of monogeneans on the gills of the cultured *Oreochromis niloticus* (n = 21) (*open bar*) and red hybrid tilapia (n = 25) (*dark bar*) (Chi square test, bootstrap 2 sample t test, *p < 0.05; **p < 0.005)

**Fig. 5:**
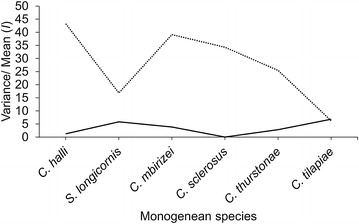
Index of dispersion, *I* (Variance/mean) of the six monogeneans on the gills of the cultured *Oreochromis niloticus* (*solid line*) and red hybrid tilapia (*dashes line*)

Figure 4 shows the prevalence and mean intensity of the monogeneans that infecting the gills of the cultured *O. niloticus* (n = 21) and red hybrid tilapia (n = 29). In the present study, significantly higher prevalence of *C. mbirizei*, *C. thurstonae* and *C. tilapiae* were observed in the cultured *O. niloticus* as compared to the cultured red hybrid tilapia, whereas *C. sclerosus* has a significant higher prevalence in the cultured red hybrid tilapia only (χ^2^ = 26.219, df = 1, p < 0.001) (Fig. 4a). In contrast, mean intensity of all the monogeneans species observed were significantly higher in the red hybrid tilapia (p < 0.05) except for *C. tilapiae*, which has significantly higher mean intensity on the cultured *O. niloticus* (t = 6.705, p < 0.001) (Fig. 4b). Bootstrap t test cannot be performed for the mean intensity of *C. sclerosus* because they were only present on the gills of the red hybrid tilapia. Most of the monogenean species adopted higher aggregative distribution in the red hybrid tilapia than in *O. niloticus* (Fig. 5), except for *C. tilapiae*, which is slightly more aggregated in *O. niloticus* (*I* = 6.82) as compared to red hybrid tilapia (*I* = 6.25) (Fig. 5).

### Spatial distribution of monogeneans on the gills of wild *Oreochromis niloticus*, cultured *Oreochromis niloticus* and red hybrid tilapia

The mean intensities of the monogeneans distributed on the gills of the wild *O. niloticus*, cultured *O. niloticus* and red hybrid tilapia were shown in Tables 3 and 4. None of the monogenean species showed a preference for either the left or the right gills of the wild *O. niloticus*, cultured *O. niloticus* and red hybrid tilapia (Mann–Whitney U test, p > 0.05) (Table 3). However, the distribution patterns of monogeneans amongst the gill arches were different among these fish hosts. In the wild *O. niloticus,* there is no significant difference in the distribution of the monogeneans amongst the four pairs of the gill arches (Table 4). In contrast, significant differences were observed in the distribution of *C. mbirizei* amongst the four pairs of gills arches from both the cultured *O. niloticus* and red hybrid tilapia (Kruskal–Wallis ANOVA, p < 0.05). In the cultured *O. niloticus*, *C. mbirizei* was found significantly more abundant on the first gill arches (L I and R I). However, *C. mbirizei* was significantly more abundant on the gill arches I, II and III of the cultured red hybrid tilapia (Kruskal–Wallis ANOVA and multiple comparison, p < 0.05) (Table 4). The numbers of *C. tilapiae* were significantly higher observed in the gill arches I, II and III of the cultured *O. niloticus* but no significant differences were observed in the red hybrid tilapia (Table 4). In contrast, the numbers of *C. halli* were significantly higher in the gill arches I, II and III in the cultured red hybrid tilapia but no significant difference was observed in cultured *O. niloticus* (Table 4). In general, the mean intensities of monogeneans were the lowest in gill arches IV of both the cultured *O. niloticus* and red hybrid tilapia (Table 4).

**Table 3:** Comparison of the mean intensity of monogenean species distributed on the left and the right gill sides of the wild *Oreochromis niloticus*, cultured *O. niloticus* and the red hybrid tilapia

Host species/gill sides	Mean intensity (cl)					
	*C. halli*	*S. longicornis*	*C. mbirizei*	*C. sclerosus*	*C. thurstonae*	*C. tilapiae*
Wild *O. niloticus*						
Left	n.a.	1.00^a^ (0.00–0.00)	n.a.	n.a.	n.a.	5.48^a^ (3.00–10.68)
Right	n.a.	1.00^a^ (0.00–0.00)	n.a.	1.00 (0.00–0.00)	n.a.	3.40^a^ (2.20–5.40)
Cultured *O. niloticus*						
Left	1.73^a^ (1.27–2.09)	2.56^a^ (1.56–3.44)	3.27^a^ (2.00–4.00)	n.a.	4.63^a^ (3.38–6.38)	12.24^a^ (9.81–15.76)
Right	1.67^a^ (1.17–2.50)	1.53^a^ (1.13–2.27)	3.38^a^ (2.44–4.56)	n.a.	3.38^a^ (2.67–4.43)	13.67^a^ (10.43–17.05)
Cultured red hybrid tilapia						
Left	20.50^a^ (13.11–28.78)	9.87^a^ (6.35–15.00)	22.50^a^ (14.69–33.25)	15.00^a^ (9.00–24.25)	12.46^a^ (7.15–18.77)	1.25^a^ (1.00–1.50)
Right	17.00^a^ (10.95–25.55)	8.36^a^ (6.09–11.18)	20.75^a^ (15.56–27.13)	12.84^a^ (8.26–19.42)	11.00^a^ (7.08–16.67)	2.50^a^ (1.25–3.25)

*cl* Bootstrap bias-corrected and accelerated confidential limit, *n.a.* represents data is not available

Levels of significant difference are represented by the superscript ‘a’ (p < 0.05)

**Table 4:** Comparison of the mean intensity of monogenean species distributed on the gill arches I, II, III and IV of the wild *Oreochromis niloticus*, *O. niloticus* and the red hybrid tilapia

Host species/gill arches	Mean intensity (cl)					
	*C. halli*	*S. longicornis*	*C. mbirizei*	*C. sclerosus*	*C. thurstoane*	*C. tilapiae*
Wild *O. niloticus*						
I	n.a.	1.00^a^ (0.00–0.00)	n.a.	n.a.	n.a.	3.93^a^ (2.43–6.86)
II	n.a.	1.00^a^ (0.00–0.00)	n.a.	1.00 (0.00–0.00)	n.a.	3.58^a^ (2.32–5.37)
III	n.a.	1.00^a^ (0.00–0.00)	n.a.	n.a.	n.a.	3.50^a^ (2.00–5.71)
IV	n.a.	1.00^a^ (0.00–0.00)	n.a.	1.00 (0.00–0.00)	n.a.	3.31^a^ (1.77–7.38)
Cultured *O. niloticus*						
I	1.67^a^ (1.22–2.00)	1.80^a^ (1.20–2.50)	4.33^a^ (3.25–5.33)	n.a.	2.89^a^ (2.06–4.44)	10.05^a^ (7.95–12.86)
II	1.00^a^ (0.00–0.00)	1.60^a^ (1.10–2.40)	1.70^b^ (1.20–2.20)	n.a.	2.53^a^ (1.84–3.37)	7.95^ab^ (6.14–10.00)
III	1.20^a^ (1.00–1.40)	1.43^a^ (1.00–2.00)	1.80^b^ (1.00–3.00)	n.a.	1.93^a^ (1.40–2.40)	5.32^b^ (4.11- 6.53)
IV	1.00^a^ (0.00–0.00)	1.00^a^ (0.00–0.00)	1.56^b^ (1.11–2.00)	n.a.	1.38^a^ (1.00–1.63)	2.59^c^ (1.94–3.24)
Cultured red hybrid tilapia						
I	16.12^a^ (10.53–23.53)	5.91^a^ (4.18–8.55)	18.69^a^ (12.13–28.13)	5.12^a^ (3.53–8.29)	8.27^a^ (4.45–12.45)	2.00^a^ (0.00–0.00)
II	12.18^a^ (7.88–17.94)	6.82^a^ (4.71–10.88)	12.77^a^ (8.08–18.92)	6.30^a^ (3.93–10.07)	6.50^a^ (3.90–8.90)	1.67^a^ (1.00–2.00)
III	10.20^a^ (6.73–14.40)	6.05^a^ (4.16–8.89)	8.40^a^ (6.33–11.47)	9.00^a^ (5.61–12.89)	7.67^a^ (5.17–12.67)	1.40^a^ (1.00–1.60)
IV	7.00^b^ (4.13–15.20)	2.94^a^ (2.12–3.76)	4.43^b^ (2.57–6.79)	8.56^a^ (5.44–12.75)	4.60^a^ (3.10–7.60)	1.38^a^ (1.00–1.75)

*cl* Bootstrap bias-corrected and accelerated confidential limit, *n.a.* represents data is not available

Different superscript alphabets indicate significant difference (p < 0.05)

## Discussion

### Infection dynamics of gill monogeneans between the wild and cultured *Oreochromis niloticus*

The present study indicates that higher species richness and infection level of monogeneans were found in the cultured *O. niloticus* compared to the wild *O. niloticus*. This concurs with the finding by Ibrahim (2012) who reported that cultured fish were more likely to be infected by monogeneans parasites due to cultivation of high density of fish in aquaculture systems. Besides, low water quality in cultured pond may also increase stresses on fish and suppress their immune system, which would promote the transmission of parasites (Landsberg et al. 1998; Shoemaker et al. 2015).

Both *C. tilapiae* and *C. sclerosus* were usually considered as generalist parasites (Mendlová and Šimková 2014). However, in the present study *C. tilapiae* was the most dominant species found in the wild and cultured *O. niloticus* whilst *C. sclerosus* was found very less in the wild tilapia or even absent in the cultured *O. niloticus.* Similarly, higher intensities of *C. tilapiae* as compared to *C. sclerosus* were found in *O. niloticus* as reported by Akoll et al. (2012) in Uganda and Pantoja et al. (2012) in Brazil. The absence of *C. sclerosus* was also noted by Tombi et al. (2014) in the cultured *O. niloticus* from the Melen fish station in Cameroon.

### Infection dynamics of gill monogeneans between the cultured *Oreochromis niloticus* and red hybrid tilapia

Higher species diversity and infection level were observed in the red hybrid tilapia than that of the cultured *O. niloticus* (Table 1; Fig. 4), indicating that hybrid tilapia are more susceptible to monogenean infections. Higher infection level of *C. sclerosus* on the gills of red hybrid tilapia as compared to *O. niloticus* was also reported by Maneepitaksanti et al. (2013). Moreover, Aguirre-Fey et al. (2015) found that the red hybrid tilapia (Pargo—UNAM), composed of 50 % Florida red tilapia, 25 % Rocky Mountain tilapia and 25 % red *O. niloticus*, have higher monogenean infection rate as compared to the wild-type *O. niloticus*. Similarly, Šimková et al. (2013) reported that hybrids of Cyprinid species (*Cyprinus carpio* Linnaeus, 1785 × *Carassius gibelio* Bloch, 1782) harboured more different species of parasites than that of their parental hosts. Higher susceptibility of hybrids towards monogeneans than their parental hosts might due to the host specificity of parasites and immunity of the fish hosts (Dupont and Crivelli 1988; Rubio-Godoy et al. 2004; Šimková et al. 2013). The authors proposed that mucus of the hybrids probably possess attractive compounds derived from each of their parental host species or that perhaps immune defences present in parental mucus is weakened in the hybrids. In the present study, *C. sclerosus* showed less preference to the gills of cultured *O. niloticus* than that of the red hybrid tilapia. This is because the natural host for *C. sclerosus* is *O. mossambicus* (see Le Roux and Avenant-Oldewage 2010) and the red hybrid tilapia might possess substances that derived from its parental host. Other biotic and abiotic factors included water quality (Madanire-Moyo et al. 2012), host sex and size (Akoll et al. 2011; Ibrahim 2012; Madanire-Moyo et al. 2011; Tombi et al. 2014; Vanhove et al. 2015) may also influence the species richness and infection level of monogeneans on tilapia fish. Further experimental studies should be performed to determine the relationship between the infection levels of monogeneans and the above mentioned factors.

All the monogeneans infected both the cultured *O. niloticus* and red hybrid tilapia are commonly found in these two species of tilapia, except for *C. mbirizei*. *Cichlidogyrus mbirizei* was first found on the gills of endemic fish, *Oreochromis tanganicae* Günther, 1894 by Muterezi Bukinga et al. (2012). Later, *C. mbirizei* had also been reported from red hybrid tilapia in Thailand by Lerssutthichawal et al. (2016). The authors suggested that *C. mbirizei* might be translocated with host during fish trading. Similarly, *C. mbirizei* may be introduced into Malaysia when tilapia fish were imported for local fish farmers and this may provide opportunities for the monogeneans to transfer and infect other closely related tilapia species (Bauer 1991; Pariselle et al. 2011).

### Spatial distribution of monogeneans on gills

The present study indicates that there was no statistical difference in the distribution of monogeneans between the left and the right gills of *O. niloticus* and red hybrid tilapia. Similarly, Agos (2013), Madanire-Moyo et al. (2011) and Tombi et al. (2014) also reported that there is no significant difference in the preferences of monogeneans on both the gill sides of *Oreochromis* spp. According to Rohde (1993), the preferences of a parasite to specific site of the host may be associated with the body symmetry of the parasites. Since *Cichlidogyrus* and *Scutogyrus* are bilateral symmetry, it is very likely that the monogeneans can have equitable distribution on both sides of the gills, which have similar morphology and exposure to ventilation current.

This study revealed that same species of monogeneans probably distribute differently over the gills of different species of hosts (*O. niloticus vs* red hybrid tilapia) as well as same species of host in different conditions (wild *vs* cultured *O. niloticus*) (Table 4). Our results also show that fish species with higher mean intensity and higher aggregation index may have higher chances to show preferences for specific gill arches (Table 4). For example, the numbers of *C. halli* were significantly higher in first three gills of red hybrid tilapia, which have higher mean intensity and aggregation, but not in both the wild and cultured *O. niloticus*. Higher aggregation distribution of the monogeneans on the gills of red hybrid tilapia facilitates the opportunities for mating and led to higher infection level in fish (Tombi et al. 2014). The present study indicates that the first two to three gills were mostly infected by all the species of monogeneans, except for the monogeneans in the wild *O. niloticus* that distributed randomly over the four pairs of gills arches. Some researchers proposed that higher preferences of parasites on median arches II and III are due to two main factors: respiratory water currents and gill surface area (Gutiérrez and Martorelli 1999; Paling 1968). The authors suggested that greater respiratory water current flowing through the gills will facilitate the settlement of these parasites. However, further study is required to determine the factors affecting the distribution of *Cichlidogyrus* and *Scutogyrus* on the gills of fish hosts. In the present study, the gill arch IV was the least infected by monogeneans in most of the cases. This may be due to the fact that the gill arch IV has the smallest colonised surfaces area and the lowest number of gill filaments as compared to the first three gill arches (El-Naggar and Reda 2003; Gutiérrez and Martorelli 1999; Madanire-Moyo et al. 2011; Tombi et al. 2014).

## Conclusion

In the present study, we revealed that same species of monogeneans have different infection dynamics over the gills of different species of tilapia and same species of tilapia kept in different conditions. In general, the cultured red hybrid tilapia have a higher monogenean infection rate than those of wild tilapia and cultured *O. niloticus*. Monogeneans prefer to harbour on the gill arches I and II but have no preference on the left or right side of the gills. The information obtained may provide strategies in aquaculture management to reduce potential economic losses of tilapia caused by parasitic infection.
